# Unsupervised classification of Blanding’s turtle (*Emydoidea blandingii*) behavioural states from multi-sensor biologger data

**DOI:** 10.1371/journal.pone.0314291

**Published:** 2024-11-25

**Authors:** Kelton Adderley-Heron, Patricia Chow-Fraser

**Affiliations:** Department of Biology, McMaster University, Hamilton, Ontario, Canada; MARE – Marine and Environmental Sciences Centre, PORTUGAL

## Abstract

Classifying animal behaviors in their natural environments is both challenging and ecologically important, but the use of biologgers with multiple sensors has significantly advanced this research beyond the capabilities of traditional methods alone. Here, we show how biologgers containing an integrated tri-axial accelerometer, GPS logger and immersion sensor were used to infer behavioural states of a cryptic, freshwater turtle, the Blanding’s turtle (*Emydoidea blandingii*). Biologgers were attached to three males and five females that reside in two undisturbed coastal marshes in northeastern Georgian Bay (Ontario, Canada) between May and July 2023. Raw acceleration values were separated into static and dynamic acceleration and subsequently used to calculate overall dynamic body acceleration (ODBA) and pitch. The unsupervised Hidden Markov Model (HMM) successfully differentiated five behavioural states as follows: active in water, resting in water, active out of water, resting in water, and nesting. Overall accuracy of the classification was 93.8%, and except for nesting (79%), all other behaviours were above 92%. There were significant differences in daily activity budgets between male and female turtles, with females spending a greater proportion of time active out of water, and inactive out of the water, while males spent a greater proportion of time active in water. These differences were likely a result of large seasonal life-history requirements such as nesting and mate finding. Accurate classification of behavioural states is important for researchers to understand fine-scale activities carried out during the active season and how environmental variables may influence the behaviours of turtles in their natural habitats.

## Introduction

Observing animal behaviour in their natural habitat is increasingly important in ecological research and behavioural ecology. Many investigators have classified different behaviours of terrestrial animals whose movements and behaviours can be filmed and/or observed visually in the wild [1]; however, studies on cryptic species are rarer, presumably because of logistical difficulty in observing them frequently or continuously when they are well camouflaged, burrowing in substrate, or under water. For endangered freshwater turtle species like the Blanding’s turtle (*Emydoidea blandingii*), individuals can stay submerged in wetlands for many hours or days during the summer, except when they surface for air. Traditional methods of collecting behavioural information *in situ* on freshwater turtle species are limited to coarse-scale data such as inferring behaviour from relocation points [2], ambient temperature loggers [3], visual observations when relocated with radio-telemetry [4] or during visual encounter surveys [5]. These established methods either capture only short periods of behaviour (since continuous capture would be prohibitively expensive and labour-intensive) or they possess limitations that prevent the continuous capture of data (e.g., GPS loggers are ineffective underwater due to the inability to receive satellite signals).

In recent years, animal-borne data-loggers with multiple integrated sensors (hereafter called biologgers), have been used to collect biological and environmental data at biologically relevant timescales [6]. Devices integrated with inertial measurement units (IMUs) such as accelerometers have provided a mechanism to capture large amounts of unannotated data. Accelerometers provide information on an individual’s body position, movement, and proxies for energy expenditure [7], while additional sensors can provide environmental information such as location (through GPS), temperature, pressure, or salinity [8]. Distinct behaviour or behavioural states (broad category representing multiple distinct behaviours) can be classified through metrics derived from the accelerometer alone, or in combination with additional sensor data.

There are several existing methods to extract behaviours or behavioural states from sensor data. Supervised computer models such as decision trees or random forest require large, labeled training databases to derive thresholds or cutoffs for each state and such an application requiring large training datasets of accelerometer data for free-ranging freshwater turtle species has recently been published [9–11]. Alternatively, unsupervised methods (k-means clustering, hidden Markov models) use complex algorithms to find patterns in data of biologically relevant behaviours or make inferences from behavioural states. To our knowledge, no published study has yet used such an unsupervised method to classify behavioural states of freshwater turtles.

Hidden Markov models (HMMs) are a state-switching time series model that can be implemented in an unsupervised or supervised context [12]. While HMMs have been mainly implemented to explore different behavioural states of GPS data, many studies have recently used them to classify accelerometer data [13, 14]. Hidden Markov models have performed similarly to other classification methods on many species [15]; they provide unique mechanisms to test effects of predictor variables on state transition probabilities and capture serial autocorrelation [12]. HMMs can incorporate different types of data as well as behavioural realism, thus allowing for more inferences to be made about complex ecological relationships.

Freshwater turtles like Blanding’s turtles may spend four to six months of the year overwintering, depending on local climatic conditions. After they emerge from overwintering, they exhibit “active” behaviours that include swimming, digging/nesting, walking, foraging as well as periods of “inactivity” such as predator avoidance, basking and resting. Given the semi-aquatic nature of this species, their active and inactive behaviours can occur both in aquatic and terrestrial environments. For example, swimming and foraging would be active behaviours in the water, while walking would be an active behaviour on land. Nesting is an activity engaged in only by gravid females, who use their hind legs to dig nests at an angle, followed by a short resting period. Given large differences in the pattern of body movements during periods of activity and inactivity, we wanted to determine if an unsupervised HMM could be used to classify Blanding’s turtle behaviour into five broad behavioural states: “Active in water”, “Active on land”, “Inactive on land”, “Inactive in water” and “Nesting”. We hypothesize that female Blanding’s turtles allocate a greater proportion of their time to being active out of water compared to males, as nesting activities demand extended terrestrial movement to locate suitable nesting sites. To our knowledge this is the first attempt to use an unsupervised method to classify behaviours of freshwater turtles during the active season.

## Materials and methods

### Study site

We conducted our study in 2023 during pre-nesting and nesting season (between May and July) in two coastal marshes on an island in the McGregor Bay archipelago in northeastern Georgian Bay, Ontario, Canada. The turtles’ core wetlands are a coastal marsh dominated by cattail (*Typha sp*.) accompanied by various sedge species. Upland habitat was largely comprised of granitic rock outcrops dominated by moss and juniper (*Juniperus sp*.*)* and mixed forests dominated by pine (*Pinus sp*.) and poplar (*Populus sp*) species. A more detailed description and map of the region can be found in a parallel study [16].

### Data collection

We initially captured turtles by using baited hoop-net traps or by hand during visual surveys in wetlands with known Blanding’s turtle populations. Upon capturing an individual, we used calipers to measure its straight carapace length (SCL), carapace height, and plastron length (to the nearest centimeter); we also used a hanging scale to weigh it (to nearest gram) and marked it by filing a combination of notches into marginal scutes [17]. After cleaning and lightly scouring the rear costal scute on the turtle carapace with sandpaper and alcohol, we attached very high frequency (VHF) radio transmitters (AI-2F, Holohil, 20g) and a multi-sensor biologger (AxyTrek, Technosmart, 10g) using a combination of epoxy glue (JB Waterweld; Permatex 5 Minute Epoxy). We weighed each individual before and after attaching the devices to ensure the total attachment weight did not exceed 5% of its body mass. We used radio transmitters to relocate the individual for device removal.

The AxyTrek biologgers included a tri-axial accelerometer, global positioning system (GPS) logger, and ambient sensors that measure water pressure, proxy conductivity (analog sensor), and temperature. The AxyManager software (TechnoSmart) was used to both configure the biologgers for deployment and to offload data. We configured the GPS logger to take a relocation every four hours when the turtle was out of the water, and the accelerometers to operate at a frequency of 10 Hz (10 readings per second), and 8-bit storage with a G-force range of ± 2 g. We configured the temperature, pressure, and conductivity sensors to record at intervals of 1 Hz (once per second). To extend battery life, we configured the conductivity sensor to disable the GPS device when the analog signal values dropped below a threshold of 500 V so that it would not waste battery searching for satellites when the turtle was immersed in water. Prior to releasing the turtle, we turned on the unit by passing a magnet over a magnetic switch on the side of the device.

### Quantifying movement parameters from sensor data

We pre-processed all sensor data in R-4.3.2 [18] using RStudio [19] with customized scripts. We used a weighted average over a 91-second window to smooth out the raw acceleration values (x, y, and z) to produce separate “static” and “dynamic” data that reflected changes in the body angle and body motion. The 91-second window was used because the dominant stroke frequency calculated for Blanding’s turtles was 91 seconds [9, 20]. Dynamic acceleration of each axis was calculated by subtracting the static acceleration from the total acceleration (see Table 1). The overall dynamic body acceleration (ODBA; [20]), a common proxy of body motion and energy expenditure, was calculated by summing the absolute dynamic acceleration from each axis [7]. We also calculated pitch from the acceleration data using the equation from [15] as shown in Table 1.

**Table 1:** Movement parameters calculated from the raw acceleration data.

Metric	Label	Equation	Description
Static Acceleration	Sx, Sy, Sz	$\frac{\sum X}{n},\frac{\sum Y}{n},\frac{\sum Z}{n}$	Average acceleration in each axis, calculated over a 91s moving window [9].
Dynamic Acceleration	Dx, Dy, Dz	Sx−X,Sy−Y,Sz−Z	Residual acceleration in each axis.
Overall Dynamic Body Acceleration	ODBA	(|Dx| + |Dy| + |Dz|)	A proxy for movement and energy expenditure [7].
Pitch	Pitch	${tan}^{-1}\left(\frac{Sx}{\sqrt{S_{y}^{2}+S_{z}^{2}}}\right)*\frac{180}{\pi }$	Vertical orientation of the body angle [15].

Subsequent summary statistics were calculated over a 30-second fixed window (max, mean, interquartile range, and variance) for the hidden Markov model classification.

We summarized movement parameters along the 10 Hz sensor time series within 30-second windows. The 16 summary statistics for each movement parameter included the maximum (max) and mean values, interquartile range, variance, and standard deviation (SD) of the means. Although HMMs allow for an infinite number of parameters to be included, additional parameters increase computational complexity and time. Hence, parameters to be included should be carefully chosen and reflect prior knowledge of the species and their behaviours [13]. The three parameters we selected for inclusion in the HMM were immersion in water (given that this is a semi-aquatic species), variance in pitch (to reflect changes in body angle), and mean ODBA (to reflect body motion intensity). These were used to classify five behavioural states during a portion of the active season (May to July): 1) being active in water (e.g. diving, surfacing, swimming), 2) being active out of water (e.g. walking on land), 3) being inactive in water (e.g. resting), 4) being inactive out of water (basking on rocks/logs etc) and 5) nesting behaviours (only for females).

### Unsupervised classification with HMM

We employed the "momentuHMM" R package [12] to fit the HMMs. Hidden Markov models require a large quantity of data captured at regular intervals (in this case, sequential 30-second intervals); however, due to battery depletion followed by reactivation upon recapture, the data were not always collected continuously, and therefore, there were periods of time with no data. Following the lead of [21], we associated each period of continuous data to an individual turtle and used its ID code as a covariate in the model’s initial distribution. We transformed the raw data for each parameter prior to reviewing probability distributions and assigning starting values for each of the three parameters selected for inclusion in the HMM. The ODBA and variance in pitch were log-transformed to reduce skewness in the data [22], while the raw analog sensor values for the immersion in water parameter were converted into a binary variable (1 if < 500 V; 0 if > 500 V; see Table 2). Both the ODBA parameter and the variance in pitch were modeled with a normal probability distribution while immersion in water was modelled with a Bernoulli distribution due to its binary nature.

**Table 2:** Starting values for the behavioural states (state-dependent probability distribution of movement parameters) used in the unsupervised hidden Markov model to classify behaviour of Blanding’s turtles (*Emydoidea blandingii*).

Variable	Distribution	Statistic	Behavioural States				
			Active in water	Active out of water	Inactive In Water	Inactive out of water	Nesting
VarPitch	Normal	Mean	-0.5	-0.5	-6	-6	0.2
		SD	3	2	3	2	1
ODBA	Normal	Mean	-1	-0.5	-2	-2	-0.5
		SD	0.8	1	0.5	0.5	0.2
Immersion in water	Bernoulli	Prob	1 − (1 × 10^−12^)	1 × 10^−12^	1 − (1 × 10^−12^)	1 × 10^−12^	1 × 10^−12^

Variance in pitch (VarPitch) and mean overall dynamic body acceleration (ODBA) were averaged over a 30-second window and then log_10_ transformed to reduce skewness. Starting values for Immersion in water were adapted by those of diving birds[15]. Prob = Probability. SD = Standard Deviation.

We constrained the transition probability matrix (TPM) to prevent certain transitions between states that were not ecologically meaningful. For example, we ensured transitions did not occur between the “inactive in water” state and “nesting” state since females need to travel in the water (active in water) and then on land (active out of water) before they can exhibit nesting behaviour. Similarly, we prevented the “nesting” state from being followed immediately by the “active in water” state, the “inactive in water” state from being immediately followed by the “active out of water” state, and the “inactive in water” state from being followed immediately by the “inactive out of water” state. Additionally, the “nesting” state could not immediately follow “active in water” state, the “active out of water” state could not immediately follow “resting in water” state, and “inactive out of water” could not immediately follow the “active in water” state. Finally, we added a covariate in the TPM and the initial distribution labeled “gravid,” reflecting the reproductive status of the turtle (either gravid or not-gravid, including males). This covariate allowed us to restrict the possibility of non-gravid female and male turtles from transitioning to or from the nesting state. This functionally means we fitted a 5-state HMM on gravid female turtles and a 4-state HMM on all other reproductive classes. The most likely behavioural states were determined with the Viterbi algorithm [12].

### Validation of the HMM

**Fig 1:**
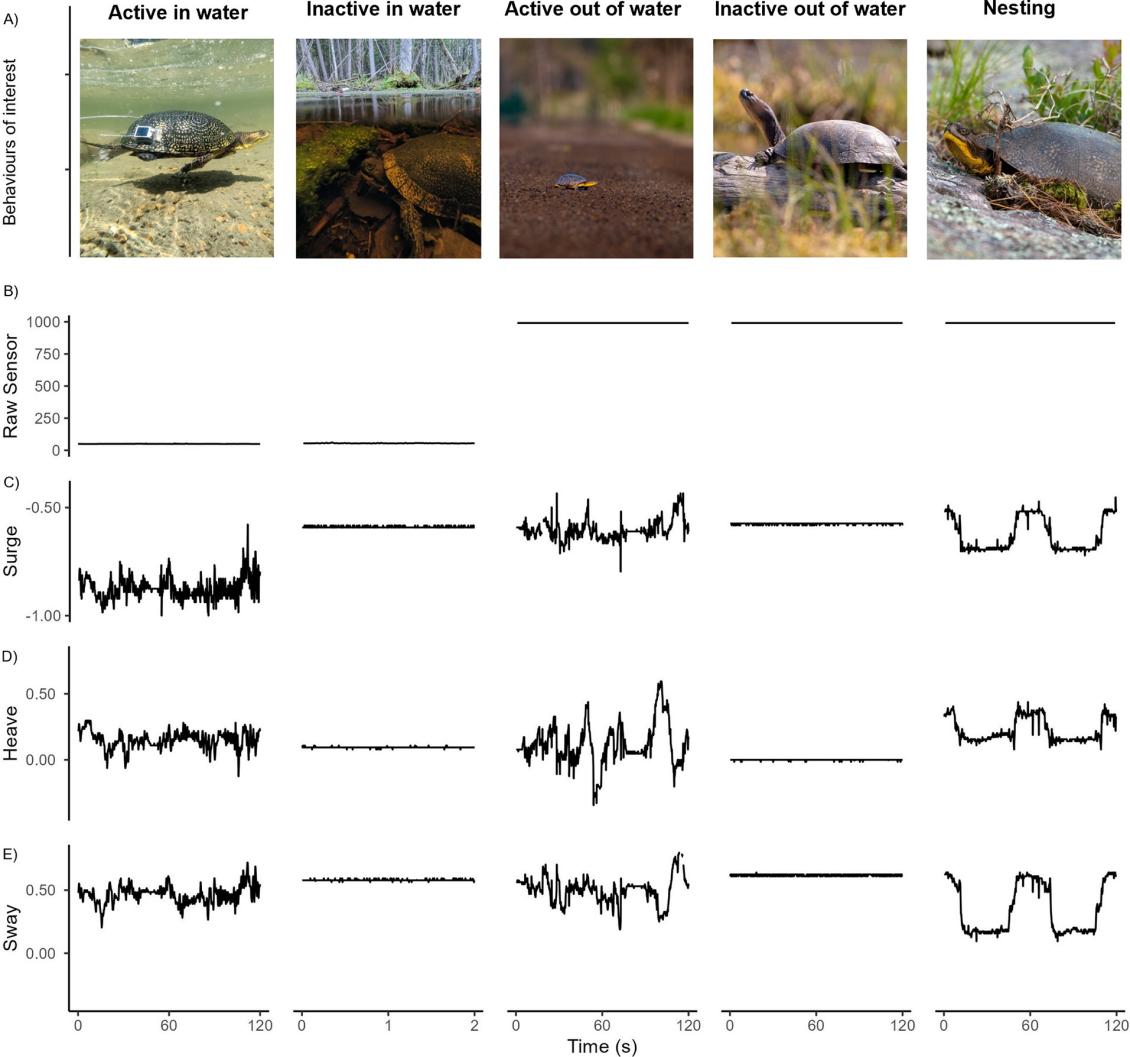
Two-minute snapshot of raw data for the five behavioural states of interest. (A) Photo of animals in each state; (B) Discrimination of in water vs out of water according to raw analog sensor data (In water threshold < = 500 V); (C) Raw surge values representing movement along the longitudinal axis (g); (D) Raw heave axis representing movement along the vertical axis (g); (E) Raw sway values representing movement along the lateral horizontal axis (g).

To validate the HMM results, we used either a smartphone camera (Google Pixel 5) or a DSLR camera (Canon 70D, 15–600 mm Sigma lens) to record contemporaneous videos of turtles wearing the biologgers in the field. The distance and timing for each recording varied depending on the ease of recording and type of behaviour expressed. For example, nesting behaviours were often captured at a distance of >15 m using a DSLR camera with a 150–600 mm telephoto lens, while active in water recordings were captured at a shorter distance with a smartphone. After viewing each video in the lab, we determined the dominant behavioural state of the individual during each 30-second segment and annotated the video using BORIS (V. 8.20.4; event logging software [23]). We omitted all instances when turtles appeared disturbed by the presence of the researcher. We only had a limited number of video recordings because it was difficult to make these recordings without disturbing the turtles. Therefore, for each individual we additionally randomly selected fifty 30-second segments and used the corresponding patterns of sensor data to classify behavioural states. This “expert-driven” method was used successfully by previous studies [13] to estimate the “true” behavioural states from sensor data without independently observed behaviour from video cameras or other methods. We have confidence in the expert-driven classification method because in this study, we focused on broad behaviours that can be directly observed and that had very distinctive signal patterns (Fig 1). We constructed a confusion matrix relating the HMM-inferred states to the annotated behavioural states using the *caret* package in R [24]. The model accuracy was determined by calculating the percentage of observations in which HMM-inferred states matched the true annotated states.

### Activity budgets

We delineated durations of continuous HMM-inferred behavioural states and determined the average of time allocated to each behavioural state by the eight turtles in the study. We also calculated the daily activity budgets (proportion of time spent in each state daily) for each sex to assess differences between sexes in movement behaviours. To assess differences in daily proportions, we fitted a mixed-effects beta regression model using the glmmTMB package in R [25]. In this model, the response variable was the proportion of time spent in each state, with sex and state included as fixed effects, and a random intercept for each individual to account for repeated daily measures. To evaluate differences in average bout duration for each state between sexes, we first assessed the data for departure from normality with the Shapiro-Wilks test. Depending on the results, we then used either a Wilcoxon rank sum test (non-parametric) or a Student’s T test (parametric) to determine significant differences between sexes. For comparisons between sexes, we combined both non-gravid and gravid females because of the limited number of non-gravid females.

### Ethics statement

Animal use and data collection was authorized by McMaster University Animal Use Protocol (22-07-27), and an Ontario Ministry of Natural Resources and Forestry Wildlife Scientific Collector’s Authorization (#1097649).

## Results

The eight biologgers recorded a total of 27,058,440 s (7516 h) of tri-axial accelerometer and immersion data for individuals across both sexes (5F, 3M). The dataset resulted in 270,584,400 accelerometer data points summarized into 901,948 30-second windows for each parameter of the HMM input. Each device recorded on average 3,382,305 ± 2,017,098 s (939 ± 560 h) of data (Table 3). Data for turtles MCG_005 and MCG_013 were only recorded for a brief period because the devices malfunctioned and unexpectedly shut off shortly after deployment and could not be reactivated. Another device on turtle MCG_016 recorded continuously for 691,200 s (8 days) before it shut off but was reactivated with a magnet during a subsequent recapture lasting an additional 20 days. The remaining five devices recorded data continuously during the entire study period without being turned off, with the longest continuous recording of 5,370,900 s (62.2 days) by turtle MCG_001 and the shortest of 2,075,070 s (24 days) by turtle MCG_003.

**Table 3:** Description of the eight Blanding’s turtles (*Emydoidea blandingii*) used in this study, conducted in McGregor Bay, Ontario, Canada.

TagID	Sex	Deployment Duration (s [h])	Deployment Start Date	Deployment End Date	Gravid
MCG_001	Female	5.3709e6 [1,493]	2023-05-09	2023-07-10	No
MCG_002	Male	5.148e6 [1,430]	2023-05-09	2023-07-08	N/A
MCG_003	Female	2.074e6 [576]	2023-05-31	2023-06-24	Yes
MCG_005	Female	9e5 [250]	2023-05-09	2023-05-19	No
MCG_006	Male	4.788e6 [1,330]	2023-05-17	2023-07-11	N/A
MCG_013	Male	6.876e5 [191]	2023-05-09	2023-05-17	N/A
MCG_016	Female	2.722e6 [756^a^]	2023-05-09	2023-06-24	Yes
MCG_017	Female	5.3496e6 [1,486]	2023-05-09	2023-07-10	Yes

Deployment duration was rounded to the nearest hour. N/A = Not applicable.

^a^ Unit was off for a 14-day period between 2023-05-17 and 2023-05-31

Throughout the study period, turtles were relocated and recorded within various habitats, including cattail marshes, the coastal zone, sedge marshes, and rock outcrops. Turtles were observed often basking in the coastal zone and marsh habitats either on the rock coastline or beaver (*Castor canadensis*)/muskrat (*Ondatra zibethicus*) lodges. Turtles were observed successfully foraging for small fish within the coastal zone. Three female Blanding’s turtles, identified as gravid during early June, made multiple nesting attempts between June 20^th^ and June 24^th^. These turtles left their core wetland and either accessed a rock outcrop within 150 m of their wetland (straight-line distances from wetland’s edge to suspected nesting site; 76 m for MCG_003 and 113 m for MCG_017) or, in one instance one individual (MCG_016) travelled both terrestrially and swam to a separate island approximately 400 m away from their core wetland.

### Accuracy assessment of HMM

**Fig 2:**
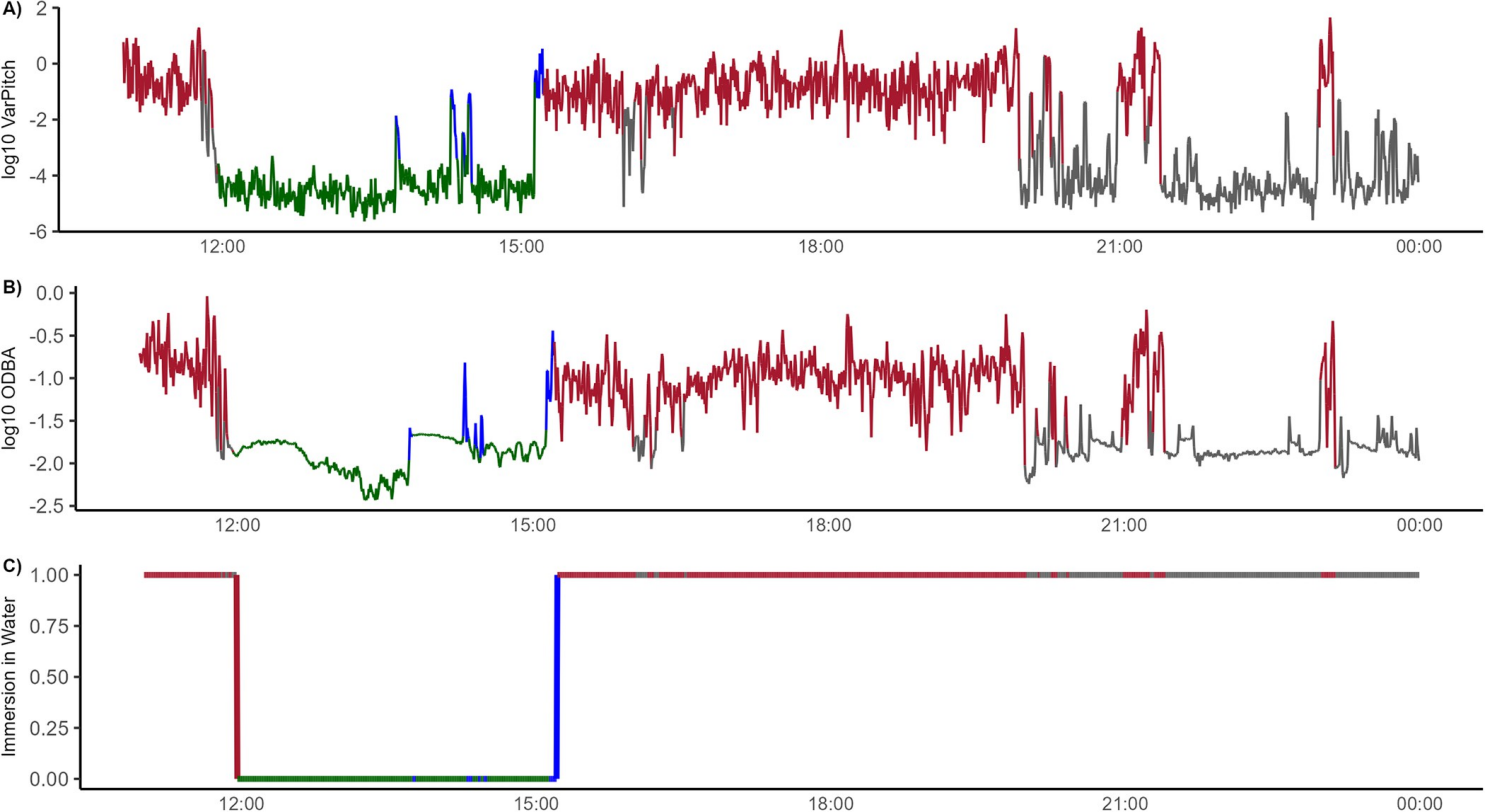
Example of classifed behavioural states inferred from unspervised 5-state hidden Markov model for turtle MCG_017 from 2023-05-15 11:00:00 to 2023-05-16 00:00:00). (A) Log_10_ variance in pitch (VarPitch) (B) Log_10_ overall dynamic body acceleration (ODBA) (C) Imersion status (threshold value of 500 V used to differentiate between “immersion in water”). Color corresponds to behavioural state (grey = inactive in water; red = active in water; blue = active out of water; green = inactive out of water).

Based on the three movement parameters (ODBA, Immersion in water, and VarPitch), the HMM classified sensor data (Fig 2) into five behavioural states with an overall accuracy of 93.8% (Table 4). Accuracies associated with being active or inactive out of water were 100%, while being active or inactive in water were slightly lower (97.5% and 92.2%, respectively); by contrast, nesting behaviours had the lowest accuracy of only 79% (Table 4).

**Table 4:** Confusion matrix to assess accuracy of the inferred behavioural states classified by the hidden Markov model (HMM) compared with the manually classified behavioural states.

		HMM Inferred				
		Active in water	Active out of water	Inactive in water	In active out of water	Nesting
**Manually Classified**	**Active in water**	97.5%	0.0%	7.8%	0.0%	0.0%
	**Active out of water**	0.0%	100%	0.0%	0.0%	21.0%
	**Inactive in water**	2.5%	0.0%	92.2%	0.0%	0.0%
	**Inactive out of water**	0.0%	0.0%	0.0%	100%	0.0%
	**Nesting**	0.0%	0.0%	0.0%	0.0%	79.0%

### Comparison of activity budgets

**Fig 3:**
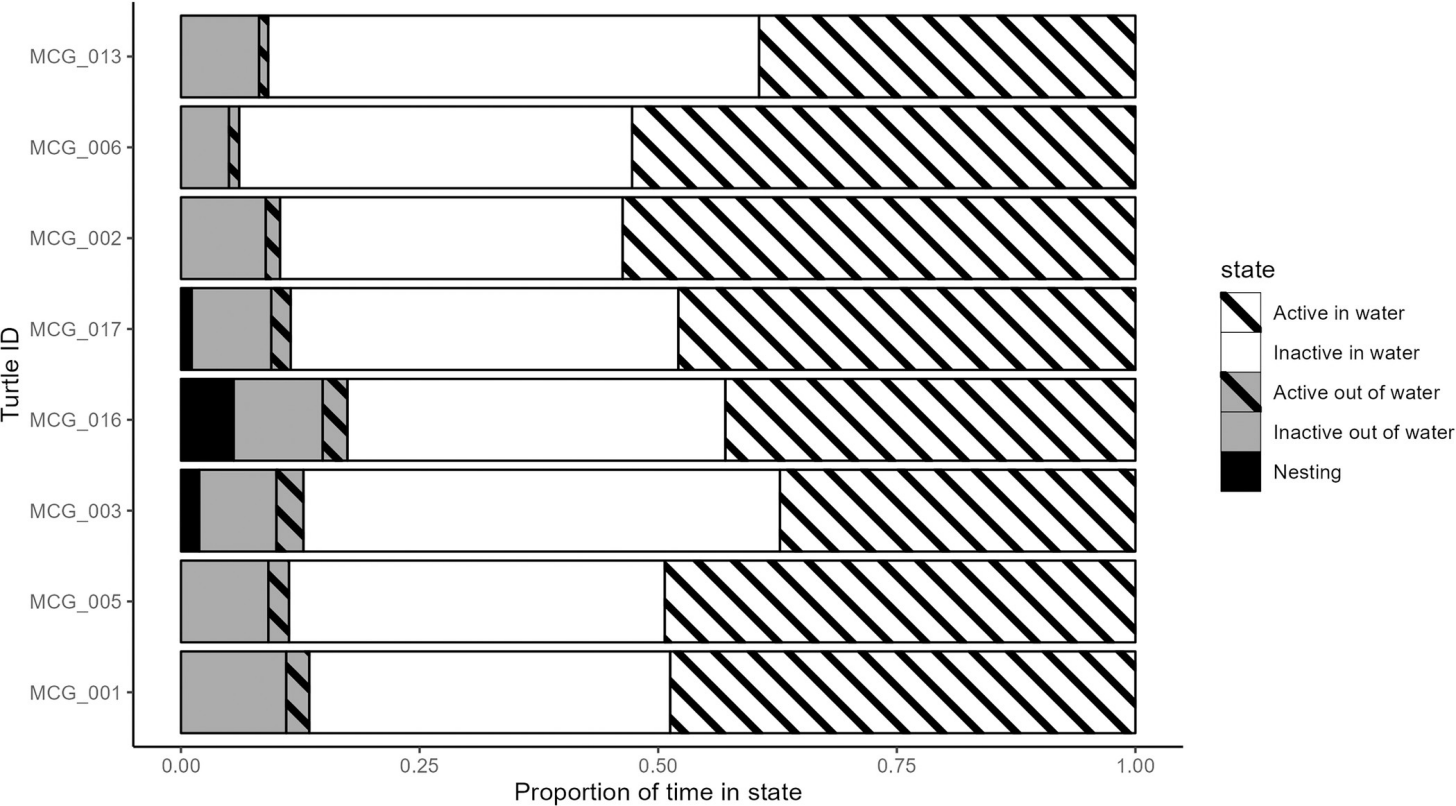
Total activity budgets (proportion of time in each state across whole deployment duration; see Table 3) for each individual Blanding’s turtle constructed from hidden Markov model-inferred states.

Throughout the entire sampling period, turtles spent more time in water than out of water, with individuals spending slightly more time being inactive in the water than being active in the water (46 ± 5% vs 41 ± 5%; Fig 3). On average, males spent significantly more time each day in water than did females (proportion of 0.53 ± 0.17 vs 0.46 ± 0.17, respectively) (p < 0.05; Table 5), while females spent a significantly higher proportion of time each day being active out of water than did males (0.02 ± 0.03 vs 0.01 ± 0.02, respectively) (p < 0.01; Table 5). There was an additional significant difference between sexes for proportion of time spent inactive out of water (0.06 ± 0.13 vs 0.09 ± 0.13 for males and females, respectively) (p < 0.01), but not for time spent inactive in the water (0.39 ± 0.15 vs 0.40 ± 0.15 for males and females respectively) (p > 0.05; Table 5).

**Table 5:** Activity budgets of males and females residing on islands in McGregor Bay Ontario.

	Males		Females		Sex Comparison	
Behavioural State	Prop	Duration (s [min])	Prop	Duration (s [min])	Prop (P value)	Duration (P value)
Active in water	0.53 ± 0.17	1121.8 ± 4100.6 [20.4 ± 68.3]	0.46 ± 0.17	958 ± 2703.4 [16.0 ± 45.1]	<0.05	<0.05
Inactive in water	0.39 ± 0.15	961.5 ± 1850.8 [16.0 ± 30.8]	0.40 ± 0.15	929 ± 1922.6 [15.5 ± 32]	0.49	0.88^a^
Active out of water	0.01 ± 0.02	217.7 ± 323.4 [3.6 ± 5.4]	0.2 ± 0.3	188.6 ± 310.2 [3.1 ± 5.2]	<0.01	0.28
Inactive out of water	0.06 ± 0.13	1811.4 ± 4035.7 [30.2 ± 67.3]	0.09 ± 0.13	1386.3 ± 2511.7 [23.1 ± 41.9]	<0.01	0.17
Nesting	-	-	0.05 ± 0.01	1039.3 ± 2058.1 [17.3 ± 34.3]	-	-

Mean daily proportion of time individuals spent in each behavioural state (prop) and mean bout duration inferred from the hidden Markov model. P values of <0.05 indicate significant differences between sexes according to statistical tests performed. If the bout duration data deviated from assumptions of parametric tests, we used a Wilcoxon rank sum test; otherwise, we used a t-test to compare between sexes.

^a^ non-normal distribution

On average, active bouts on land (i.e. out of the water) were shortest, lasting only 195 ± 314 s (3 ± 5 min), while nesting bouts averaged 1039 ± 2058 s (17 ± 34 min; maximum duration was 286 minutes). Inactive periods on land, however, were the longest, averaging 1502 ± 3008 s (25 ± 50 min), with one individual having an inactive period lasting up to 58,560 s (16.3 h). Active and inactive periods in water had similar durations of 1054 ± 3286 s (17 ± 55 min) and 941 ± 1896 s (16 ± 32 min), respectively. Overall, only the duration of active bouts in water were significantly different between sexes, where males performed on average longer bouts of activity in the water than did females (1121.8 ± 4100.6 s, 958 ± 2703.4 s respectively) (Student’s *t* test; p < 0.05; Table 5).

## Discussion

We successfully discerned five dominant behavioural states of a semi-aquatic freshwater turtle using data from a multi-sensor biologger and an unsupervised HMM. By adding a biological variable to indicate gravid or non-gravid status, we eliminated the possibility that activities associated with males or non-gravid females could be classified as “nesting” behaviours. Despite the high overall accuracy of 93.8%, accuracy for the nesting behavioural state was only 79%. We attribute this lower accuracy to our choice of 30-second window, which is too short to capture the entire behavioural state, which includes rhythmic movements of the gravid female while they use their hind legs to dig at an angle, followed by a brief resting period (Fig 1). Previous research [13] has discussed how the use of an inappropriate time window to summarize data could lead to misclassifications. We were able to visually distinguish accelerometer data associated with nesting behaviour from those of other behaviours when we used a longer time window; however, increasing the time window would have reduced classification accuracy for all other states because “activity” would have overshadowed short bouts of “inactivity”. Although use of sliding windows [26] may help solve this problem, it may lead to other problems related to handling much larger datasets and unreasonably long processing times and is therefore not a practical solution.

Although all nesting attempts observed in the field were correctly classified as nesting, some overland movements with similar variance in pitch and ODBA were incorrectly classified as nesting (Table 4). A posterior correction could be applied to the classified data to further reduce errors by incorporating spatial information to mask out areas that do not have typical nesting habitat. Another option is to use additional covariates in the model to restrict transitioning to nesting behavioural state outside of the nesting season and where there are unsuitable habitat types. We did not apply these posteriori corrections to our data since our primary goal was to test the capability of the HMM for classifying behavioural states for the Blanding’s turtle.

While we were able to distinguish between the dominant behavioural states, limitations in our data collection and classification method prevented us from distinguishing between more nuanced behaviours (e.g. surfacing and foraging events). First, the pressure sensor was an unreliable method of distinguishing surfacing events in most cases, as the turtles spent a large portion of their time in shallow areas or near the surface of the water, where the sensor’s sensitivity was insufficient. A previous study on European pond turtles (*Emys orbicularis*) used a supervised classification method to successfully capture some surfacing events overnight, when other movement is minimal [10]. Applying a similar supervised method to our data could also potentially capture nocturnal surfacing events or instances when the turtles are in deeper water. Secondly, although foraging behaviours are ecologically important, they show limited notable/consistent differences in body kinematics compared to other behaviours like being active or inactive, which limited our ability to distinguish them. Blanding’s turtles’ diet has been found to consist largely of animal matter [27] and reported observations describe them as ingesting prey when individuals probe their head into vegetation, scavenging, and some active predation [27, 28]. With the diverse diet and inconsistent predation behaviours classifying foraging behaviour would likely require a supervised training approach and a large amount of training data.

### Behavioural partitioning between sexes

We quantified differences in average daily activity budgets between males and females. On average, females spent a significantly larger proportion of their daily activity on land (out of water) and this may be attributed to their well-documented overland migrations to nest [29, 30]. In many studies, gravid females will travel long distances to find suitable nest sites to maximize the chance of hatchling survival [31]. In the Georgian Bay landscape with rock outcrops, substrate depth is often limited [32] and turtles may have to test multiple locations before they successfully nest. The larger proportion of time spent on overland travel to access nesting habitat and then selecting nest sites may explain why females spend a significantly larger proportion of their time being active out of water compared to males.

Females also spent a significantly smaller proportion of time being active in water and this may reflect the greater proportion of time that males must spend to find mates. While females continue to travel for nesting purposes, increasing their vulnerability to predation, males spend most of their time during the pre-nesting season searching for and mating with females, as well as foraging, activities that primarily take place in the water, therefore, reducing the amount of time on land [33]. Another reason why the males may be spending more time in water may be related to our study site being in an undisturbed archipelago, with most of the turtles inhabiting two coastal marshes that are in close proximity (~ 200 m apart but on the same island). A parallel study [16], confirmed that this population used habitat types according to their availability and did not exhibit positive or negative selection for any habitat class at the third-order scale. Since the core wetlands are large and in close proximity, males probably do not need to travel far to access mates and resources.

We found evidence of differences in the proportion of daily inactivity between males and females only when they were out of the water. Being ectotherms, turtles must spend long periods in the early spring being out of or at the water surface to bask to increase their body temperatures [3]. Gravid females have been reported to spend a greater percentage of time basking [3] likely due to requirements for egg development [34] and likely what is contributing to the difference in this case. As the weather warms up when females conduct overland nesting migrations, they must still periodically use aquatic habitats such as wetlands or vernal pools to hide from predators, to rehydrate, and to replenish energy reserves [4, 35]. Therefore, on average, the proportion of time each day spent being inactive in the water would not differ significantly between males and females.

While these behaviours may be explained by large seasonal life-history requirements such as nesting and mate finding, other environmental characteristics may affect behaviour patterns. An animal’s behaviour and activity patterns reflect the spatial and temporal processes that influence its ability to acquire resources. For example, spatial processes such as habitat structure and heterogeneity, variation in moisture, as well as temporal processes such as daily and seasonal fluctuations in photoperiod, ambient temperature and precipitation can all affect the distribution of resources and the ability of individuals to exploit the resource [36]. Such daily and seasonal variations in environmental conditions should influence how animals make short-term decisions on how to select suitable habitats or when to forage for food or seek shelter, while minimizing exposure to predation [37]. Integrating variables into the models that correspond to these temporal and spatial processes will help further elucidate the more fine-scale drivers of behaviour.

Understanding how Blanding’s turtles partition their behaviours in a natural, undisturbed habitat can provide insights into how disturbances may impact populations. For instance, the difference in activity outside the water, combined with the time females require for nesting and the time males spend in the water, highlights the potential for increased mortality risk for female turtles compared to males within this population. While road mortality and urban subsidized predators are not threats in this undisturbed location, future research should explore whether this pattern persists in disturbed areas, as it could help explain the male-biased sex ratios observed in other turtle populations [33].

## Conclusions

We have demonstrated the efficacy of an unsupervised HMM framework for classifying behavioural states in Blanding’s turtles using high-frequency multi-sensor data. Nevertheless, this approach was unable to accurately model more than five behaviours, and the behavioural states decoded by the model were not always aligned with biologically interpretable behaviours. Therefore, more nuanced behaviours such as foraging or surfacing events may need to be approached with a supervised classification method using the HMM-inferred states. The methods outlined in this study were only applied to a single population of Blanding’s turtles in McGregor Bay. Even so, the framework we employed could be modified for other species in other environmental contexts, provided the targeted behaviours exhibit notable differences in body kinematics. Furthermore, this framework offers a basis for investigating the drivers of behaviours and how they might be manifested temporally and spatially across various habitats and environmental conditions for the Blanding’s turtle. By improving our understanding of these behaviours and their temporal and spatial manifestations, we can inform more effective conservation strategies aimed at the specific needs of Blanding’s turtles and other similar species.
